# *Arcobacter butzleri*: Underestimated Enteropathogen

**DOI:** 10.3201/eid1202.050570

**Published:** 2006-02

**Authors:** Valérie Prouzet-Mauléon, Leila Labadi, Nathalie Bouges, Armelle Ménard, Francis Mégraud

**Affiliations:** * Hôpital Pellegrin, Bordeaux, France;; † Université Victor Segalen Bordeaux 2, Bordeaux, France

**Keywords:** Arcobacter, Campylobacter, 16S rRNA gene sequencing

## Abstract

Molecular methods applied to 2,855 strains of *Campylobacter*-like organisms received from a surveillance network of *Campylobacter* infections in France identified 29 *Arcobacter butzleri* infections. This species ranks fourth for *Campylobacteraceae* isolation and appears to have the same pathogenic potential as the other species in the genus.

Kiehlbauch et al. (*1*) originally described the species *Arcobacter butzleri*, previously named *Campylobacter butzleri* (*1*), after studying aerotolerant *Campylobacter* strains from human and veterinary sources. The genus *Arcobacter* was created in 1992 (*2*).

*A. butzleri* is found in environmental samples, and untreated water appears to be a potential source of infection. In industrialized countries, the most important source of human contamination may be food. Indeed, *A. butzleri* has been isolated in different breeding animals and is present in a great variety of retail meats, including chicken, beef, pork, and lamb, with a high prevalence in poultry (*3*).

Although the prevalence of this bacterium in animals and food specimens is well documented, including the first publication by Kiehlbauch et al. that implied this newly described species could be an important human pathogen, only a few reports of human infections are found, and most of them were published before 1995. In the Far East, Taylor et al. formally identified *A. butzleri* in 2.4% of diarrheal stool samples collected from Thai children (*4*), and more recently, 2 bacteremia cases were reported in Taiwan and Hong Kong in patients with an underlying disease (liver cirrhosis and gangrenous appendicitis, respectively) (*5**,**6*). In a South African study, 15 *A. butzleri* were identified among 3,877 *Campylobacteraceae* strains isolated from children's diarrheic stools (*7*). Reports from Europe are scarce: an outbreak of recurrent abdominal cramps in 10 patients in Italy (*8*), bacteremia in a newborn in the United Kingdom (*9*), and 2 cases of severe diarrhea in Germany (*10*). The recent publication of Vandenberg et al. from Belgium used an *Arcobacter*-selective medium for stool specimens and found that *A. butzleri* ranked fourth among *Campylobacter* spp. and *Campylobacter*-like organisms (*11*), which stimulated interest to revisit the role of *Arcobacter* spp. as an agent of enteric infection.

In this study, our goal was to investigate the prevalence of *A. butzleri* with a different approach and to describe the clinical features of *A. butzleri* infection. We used molecular methods to identify *Campylobacter*-related organisms collected from a network of clinical laboratories that do not use specific *Arcobacter*-selective medium. The results nevertheless showed that this bacterium ranks fourth among these organisms.

## The Study

The French surveillance network of human *Campylobacter* infections is composed of laboratories selected on a voluntary basis (*12*). The laboratories are randomly located throughout France; 93 are hospital laboratories, and 338 are private laboratories. They send their clinical *Campylobacter* isolates to the National Reference Center with a completed information sheet concerning the patient and epidemiologic data. The study period was from July 2002 to December 2003, and 2,855 *Campylobacter*-like strains were studied. In addition to standard phenotypic identification, specific polymerase chain reaction (PCR) assays were carried out to identify *C. jejuni*, *C. coli*, or *C. fetus* (*13*); other isolates were identified by comparing 2 sequences of 400 bp located at both extremities of a 1,100-bp fragment of the beginning of the 16S rRNA gene amplified with primers F2-16S-CHPEC (ATCCTGGCTCAGAGTGAACG) and R2-16S-CHPEC (AAGGGCCATGATGACTTGAC) with those of DNA databases by using the BLAST program (http://www.ncbi.nlm.nih.gov/blast/). An identification at the species level was regarded as correct when >99% identity was seen with only 1 species. The species distribution from stool samples was as follows: 2,114 *C. jejuni* (78.9%), 486 *C. coli* (18.1%), 36 *C. fetus* (1.3%), 27 *A. butzleri* (1.0%), 8 *C. lari* (0.3%), 4 *C. upsaliensis* (0.1%), 2 *C. hyointestinalis*, and 1 *Helicobacter canadensis*. From 177 nonstool samples, we detected 2 *A. butzleri*.

Almost the entire 16S rRNA gene, 1,450 bp, was sequenced for 9 *A. butzleri* to study its homogeneity because only 5 sequences are in the databank (accession nos. AF314538, AY621116, L14626, U34386, U34388). The intraspecies variability was only 0.4% (6/1,430 bp) among the 14 *A. butzleri* sequences. Comparison of these sequences with those of 4 *A. cryaerophilus* (AY314755, L14624, U34387, U25805), 1 *A. skirrowii* (L14625), and 1 *A. nitrofigilis* (L14627) showed that interspecies variability concerned 14 bp out of 1,430. The *Arcobacter* sequences differed considerably from those of *Campylobacter* spp., with only 86.2% identity with *C. coli* (AY621115) and 85.6% identity with *C. fetus* (AY621110), 2 species that can be phenotypically confounded with *A. butzleri*.

Twenty-seven of the 29 *A. butzleri* strains were isolated from feces of patients with gastroenteritis, whereas 1 strain was isolated from peritonitis pus and another from a blood culture specimen. The 27 *Arcobacter* strains from stools were isolated in 23 different laboratories. Most of them (n = 15) used Campylosel (bioMérieux, Marcy l'Etoile, France) incubated at 37°C for 2 to 3 days. Six used Karmali agar incubated at 37°C for 3 to 4 days. The last 2 used Butzler medium and the filtration method, respectively. None of these infections was part of an outbreak. Of the 16 patients with travel information available, only 1 indicated a recent trip, to Turkey. An associated pathogenic organism was described in 1 of 15 cases, but the nature of the associated organism is unknown. Seventeen of the 29 patients were hospitalized. Their age range was 1–89 years, with an average of 54 years. Seventeen patients (59%) were male. Detailed clinical information was obtained for 19 patients and is shown in the Table. Eighteen of 19 patients had diarrhea, including the patient with bacteremia; 11 of 15 had abdominal pain; 4 of 15 had bloody stools, including the patient with *A. butzleri* bacteremia; 2 of 14 patients vomited; and 5 of 17 patients had fever. Acute renal failure developed in 2 patients, 1 associated with pyelonephritis. Concerning the 17 other cases for which information was available, severe clinical symptoms (anorexia, weight loss, asthenia) were described for 10 patients.

**Table:** Demographic and clinical characteristics of 19 patients infected with *Arcobacter butzleri*

Patient*	Age (y)	Sex	Diarrhea	Abdominal pain	Blood in stool	Vomiting	Fever	General symptoms†	Duration of symptoms and disease course
1	88	F	+	+	–		+	+	Death linked to associated neoplasia
2	75	M	+	+	–	–	–	+	4 days. Acute renal failure (treated with amoxicillin)
4	89	F	+	–	–	–	–	+	6 weeks. Favorable progress after treatment with ofloxacin
7	76	M	+	+	+	–	–	–	5 days (treated with ciprofloxacin)
8	2	M	+	–	–	+	–	+	2 days
10	69	M	+	+		–	–	+	
11	4	M	+	+	–	–	+	+	Several recurrences in 3 months
12	72	F	+	–	+	–	–	–	3 weeks
15	89	M	+		–			–	
18	53	M	+	+	–	–	–	+	Irritable bowel syndrome
19	6	M	+	+	+	+	+	+	10 days
20	30	F	–	+	–	–	–	–	
21	68	M	+	+	+	–	+	+	
23	77	M	+				–	+	Several days. Acute renal failure (treated with ciprofloxacin)
25	1	F	+				+	+	3 weeks
26	41	M	+	–	–	–	–	+	Several days (treated with amoxicillin-clavulanate)
27	44	F	+	+	–	–	–	–	
28	70	F	+						
29	83	F	+	+	–	–	–	+	Several days

*All samples were from stool, except from patient 12, whose sample was blood. †Anorexia, asthenia, weight loss.

The duration of symptoms without treatment was variable, from 2 days to several weeks. Antimicrobial drug therapy was administered in 5 cases, and infection was eradicated a few days later (Table). In all cases, the strains were susceptible to the antimicrobial agent used (amoxicillin, ofloxacin, ciprofloxacin [twice], amoxicillin-clavulanate).

## Conclusions

During an 18-month period, 2,855 strains of *Campylobacter*-like organisms were identified at the species level by using phenotypic and molecular tools. Molecular methods permitted the identification of 29 *A. butzleri*, i.e., 1% of the strains studied and also 1% of the 2,678 isolates from stools, which makes this species the fourth most frequently isolated *Campylobacteraceae* in human clinical samples in France, before *C. lari* and *C. upsaliensis* but after *C. jejuni*, *C. coli*, and *C. fetus*. Our results agree with those of Vandenberg et al. (*11*) who also found *A. butzleri* in fourth position for *Campylobacter*-like isolates from stool specimens. However, their results were obtained in a different context: only 1 laboratory was involved for 8 years, and a specific *Arcobacter*-selective medium was used. A study conducted in Denmark by Engberg et al. (*14*), also to estimate the prevalence of *Campylobacter* spp. and related bacteria in human fecal samples, did not produce the same results; only 1 *A. butzleri* was isolated out of 1,376 samples, and no *C. lari* or *C. upsaliensis* was recovered. The differences between these 3 studies could be explained by differences in geographic distribution of the species, but the difference in isolation methods (selective agar in the Belgian study) or in identification methods (molecular methods in our study) is likely the critical point. While not optimal, *Campylobacter*-selective agars, especially Campylosel, appear to allow the growth of *A. butzleri*. For the other media, a longer incubation period seems necessary. The role of *A. butzleri* in enteric infections is not definitively proven, but its involvement is likely. In at least 14 of 15 cases, no other enteropathogen was detected. In 1 case, a bacteremia with an enteric infection was found. These data are similar to those observed with *C. jejuni* infection. Isolation of *A. butzleri* is more frequent than well-known enteropathogenic *Campylobacter* spp., e.g., *C. upsaliensis* and *C. lari*, when adequate identification is carried out. The prevalence of *A. butzleri* may be underestimated because of false identification as *C. coli* or *C. fetus*. Indeed nearly all of the strains are able to grow at 25°C, like *C. fetus*, but are resistant to cephalothin like *C. coli*. We found that nearly all of the strains were resistant to nalidixic acid (4 had an intermediary resistance) and susceptible to ciprofloxacin with the disk diffusion method. The choice of a simple method for testing these drugs allows it to be used routinely in clinical laboratories to differentiate such strains phenotypically.

Nevertheless, molecular biology is a powerful tool for diagnosis. We used a 16S rRNA gene sequencing as a global approach for strains that were not *C. jejuni*, *C. coli*, or *C. fetus*. We confirmed that the intraspecies variability of *A. butzleri* 16S rRNA gene sequences is low (0.4%), lower than that found for *C. coli* (1.5%) or *C. lari* (2.5%) (*15*), and that interspecies variability is high. This approach, which is expensive and difficult to implement, is not accessible to a routine laboratory, but simple phenotypic tests can be applied to detect these microorganisms. The combination of phenotypic tests and selective agar medium will determine the real incidence of *A. butzleri* infection, which will add evidence that *A. butzleri* is an etiologic agent of diarrhea. A determination of the prevalence of *A. butzleri* in normal stool will also help establish the prevalence of this organism in diarrheal stool.
